# *Streptococcus* *dysgalactiae* subsp. *equisimilis* from Diseased Pigs Are Genetically Distinct from Human Strains and Associated with Multidrug Resistance

**DOI:** 10.3390/microorganisms14010009

**Published:** 2025-12-19

**Authors:** Fengyang Hsu, Kayleigh Gauvin, Kevin Li, Julie-Hélène Fairbrother, Jared Simpson, Marcelo Gottschalk, Nahuel Fittipaldi

**Affiliations:** 1 Groupe de recherche sur les maladies infectieuses en production animale (GREMIP), Faculté de médecine vétérinaire, Université de Montréal, St-Hyacinthe, QC J2S 2M2, Canada; fengyang.hsu@umontreal.ca (F.H.); kayleighgauvin@gmail.com (K.G.); kevin.li.2@umontreal.ca (K.L.); julie-helene.fairbrother@mapaq.gouv.qc.ca (J.-H.F.); marcelo.gottschalk@umontreal.ca (M.G.); 2 Centre de recherche en infectiologie porcine et avicole (CRIPA), Faculté de médecine vétérinaire, Université de Montréal, St-Hyacinthe, QC J2S 2M2, Canada; 3 Complexe de diagnostic et d’épidémiosurveillance vétérinaires du Québec, Ministère de l’Agriculture, des Pêcheries et de l’Alimentation du Québec, St-Hyacinthe, QC J2S 2M2, Canada; 4 Ontario Institute for Cancer Research, Toronto, ON M5G 1M1, Canada; jsimpson@oicr.on.ca; 5 Department of Molecular Genetics, University of Toronto, Toronto, ON M5S 1A8, Canada; 6 Genetics and Genome Biology Program, The Hospital for Sick Children, Toronto, ON M5G 1E8, Canada

**Keywords:** *Streptococcus dysgalactiae* subsp. *equisimilis*, SDSE, Lancefield groups C and L *Streptococcus*, comparative genomics, *emm* gene, antimicrobial resistance (AMR), zoonoses, Canada

## Abstract

*Streptococcus dysgalactiae* subsp. *equisimilis* (SDSE) has historically been recognized as a human pathogen, yet β-hemolytic streptococci consistent with SDSE have been documented in pigs for nearly a century. To investigate the population structure of porcine SDSE and the phylogenetic relationships between swine and human strains, we characterized 41 isolates recovered from diseased pigs in Quebec, Canada (2019–2022). Infected animals spanned all major production stages and frequently presented with invasive disease, including arthritis, endocarditis, and sudden death. Core-genome phylogenetics resolved two heterogeneous porcine clades separated by long internal branches and clearly distinct from dominant human SDSE lineages. Most porcine isolates were *emm*-negative or contained structurally altered *emm* regions compared with human strains. Analysis of Lancefield antigen loci identified a predominant group C lineage and a minority group L lineage, recapitulating historical serogroup distributions described since the early-20th century. Phenotypic testing showed susceptibility to β-lactams and florfenicol but high levels of resistance to tetracycline, macrolides and lincosamides. Detected antimicrobial resistance (AMR) genes correlated well with phenotypes, and multidrug resistance was frequent. Hybrid genome assemblies revealed integrative and mobilizable elements carrying AMR determinants. Collectively, our data indicate that porcine SDSE represents a long-standing, genetically structured, host-adapted population with notable AMR potential, underscoring the need for continued swine SDSE genomic surveillance.

## 1. Introduction

*Streptococcus dysgalactiae* subsp. *equisimilis* (SDSE) is a β-hemolytic pathogen traditionally associated with human infections [1]. It most frequently expresses Lancefield group C or G and, in some cases, group A or L antigens [2,3,4]. While SDSE can asymptomatically colonize the human pharynx, skin, gastrointestinal tract, and female genitourinary tract, it may also cause severe invasive infections in individuals with compromised skin barriers or underlying conditions [1,5,6]. These infections resemble those caused by *Streptococcus pyogenes* and include skin lesions, necrotizing soft tissue infections, sepsis, and streptococcal toxic shock syndrome [1,7,8,9]. Human SDSE strains possess a diverse array of virulence factors also found in *S. pyogenes* such as the M protein, streptolysins S and O, streptodornase and C5a peptidase, as well as global virulence regulators such as the control of virulence regulon (CovRS) and the streptococcal invasive locus (*sil*) [10,11]. These determinants facilitate host colonization, tissue invasion, immune escape, and systemic dissemination during invasive disease [10].

SDSE is less well recognized as a pathogen in animals. Most research on animal-derived SDSE has focused on horses [12,13,14], whereas studies on pig isolates have been scarce [15]. SDSE can cause swine disease across all production stages, from neonatal piglets to breeding sows, presenting with clinical signs such as astasia, lameness, swollen joints, neurological symptoms, and sudden death [16,17,18,19,20,21,22]. Reported pathological findings include suppurative arthritis, endocarditis, and meningoencephalitis, and in severe cases, multifocal micro-abscesses in the heart and kidneys may be observed [17,19].

Although infrequent, reports of SDSE zoonotic transmission involving animal exposure, particularly among individuals in direct contact with pigs or raw pork, have raised public health concerns [14,23]. In addition, pig-derived strains might act as reservoirs of antimicrobial resistance (AMR), further contributing to a potential but underrecognized health threat. Preliminary studies suggest that pig-derived isolates may form host-associated lineages, but this possibility remains unresolved due to limited sampling and incomplete genomic data [16,22,24]. Indeed, there is a notable paucity of genome data for porcine SDSE with only a few porcine SDSE genomes available in public repositories as of November 2025 [25].

To clarify the relationships between porcine and human isolates, here, we tested two alternative hypotheses: (1) that SDSE from diseased pigs form a genetically distinct population with unique virulence and resistance features or (2) that they fall within the known diversity of human-associated strains. Through comparative genomics and phylogenetic analysis of 41 clinical isolates from diseased pigs in Quebec, Canada, we show that porcine SDSE isolates form a genetically distinct population. Although the swine isolates share several virulence factors with human strains, they differ in the presence of specific virulence genes—particularly within the *emm* locus—as well as in their AMR profiles.

## 2. Materials and Methods

### 2.1. Isolate Collection and Clinical Data

Our collection comprised 41 SDSE isolates obtained between 2019 and 2022 from diagnostic submissions of clinically diseased pigs from Canadian herds to the Centre de diagnostic vétérinaire de l’Université de Montréal (CDVUM) or the two Animal Health Laboratories of the Ministère de l’Agriculture, des Pêcheries et de l’Alimentation du Québec (MAPAQ). These organisms represent all SDSE porcine isolates recovered during the period (Table S1). Clinical samples included specimens from normally sterile sites, namely the brain, heart valves, spleen, liver, kidneys, joints (synovial fluid), and peritoneal fluid, as well as specimens from non-sterile sites, including cutaneous abscesses, lungs, and upper respiratory tract samples (nasal cavity and tonsils). Samples were cultured on Columbia agar supplemented with 5% defibrinated sheep blood (Thermo Fisher Scientific, Mississauga, ON, Canada) and incubated at 37 °C under 5% CO_2_ for 24–48 h. Colonies exhibiting β-hemolysis, small-to-medium size, and morphology consistent with β-hemolytic streptococci were considered presumptive β-hemolytic streptococci. Presumptive isolates were subcultured to purity and identified as “*S. dysgalactiae*” using matrix-assisted laser desorption/ionization time-of-flight mass spectrometry (MALDI-TOF MS, Microflex LT/SH mass spectrometer, Bruker Daltonics, Milton, ON, Canada). Generated spectra were compared with those of the MBT compass library 2023, ref 1829023, v13 (Bruker Daltonics). Clinical and epidemiologic metadata of isolates, including animal age, production stage, sample type, and associated lesions, were recorded when available.

### 2.2. Bacterial Growth Conditions, DNA Preparations and Whole Genome Sequencing

Isolates were grown overnight (ON) at 37 °C under 5% CO_2_ on Columbia agar plates supplemented with 5% defibrinated sheep blood (Thermo Fisher Scientific, Mississauga, ON, Canada). Several colonies per plate were next subcultured in Todd-Hewitt broth (Thermo Fisher Scientific) supplemented with 0.2% yeast extract and incubated ON under the same conditions. Genomic DNA was extracted from 5 mL of ON growth using the QIAamp DNA Mini Kit (Qiagen, Toronto, ON, Canada), as previously described [26]. DNA quantity and purity were assessed by spectrophotometry (NanoDrop, Thermo Fisher Scientific), and DNA integrity was verified by agarose gel electrophoresis prior to library preparation. DNA sequencing libraries were prepared using NEB Ultra II library preparation kits (NEB, Whitby, ON, Canada) and sequenced as paired end reads (2 × 150 bp) on an Illumina NovaSeq 6000 platform (Illumina) at the facilities of Génome Québec (Montreal, QC, Canada). Since swine-derived SDSE reference genomes were not available, to facilitate genome circularization, the genomes of two isolates (NSDE00108 and NSDE00111) were additionally sequenced on an Oxford Nanopore Technologies (ONT) MinION instrument at the facilities of Cambridge Technologies (Worthington, MN, USA).

### 2.3. Species Confirmation, Multilocus Sequencing Tying and emm Typing

Since MALDI-TOF MS does not permit subspecies differentiation, we used short-read genome data and Kraken v2 for taxonomic confirmation of the isolates to SDSE and differentiation from *S. dysgalactiae* subsp. *dysgalactiae* [27]. Multilocus sequencing typing (MLST) information was acquired from short-read data using SRST2 v0.2.0 [28] and the SDSE pubMLST database (https://pubmlst.org/, accessed on 15 November 2025) [29,30]. Clonal complexes (CCs) and singletons were defined at the single-locus variant (SLV) level using goeBURST analysis in PHYLOViZ 2.0 [31]. We determined *emm* types using an *emm* typing pipeline [32] and the *emm* sequence database curated by the United States Centers for Disease Control and Prevention.

### 2.4. Genome Assembly, Annotation, Mobile Element Detection, Comparative Genomics, and Phylogenetic Analyses

To circularize the genomes of selected isolates, we combined Illumina short-read and ONT long-read data with Unicycler with default parameters [33]. Final hybrid assemblies were manually inspected for circularity by alignment dot plots. Genome annotations were carried out with Prokka v1.14.6 [34]. Core and accessory genes of the circularized porcine isolates and the human genome MGCS36044 were identified by clustering homologous genes with Roary v3.13.0 [35]. Identification of mobile genetic elements (MGEs) such as integrative and conjugative elements (ICEs), integrative mobilizable elements (IMEs), and associated recombinases, was performed using MobileElementFinder v1.0.3 [36] and ICEscreen v1.3.3 [37]. Prophage regions were identified using the PHASTEST v3.0 [38]. For isolates with only short-read data, we performed de novo assemblies using the A5-MiSeq pipeline [39]. Core-genome phylogenetic analysis was performed as follows: we first identified single-nucleotide polymorphisms (SNPs) using the Snippy v4.6.0 algorithm [40] relative to the reference SDSE swine strain NCTC6403 (GenBank Accession number: NZ_LR594046) as a reference genome sequence (Table S2). For the purposes of comparative analysis, we incorporated the genomes of seven human SDSE organisms, selected to represent the range of genetic diversity observed among human SDSE [41], as well as six additional SDSE genomes from swine (NCBI’s BioProject: PRJEB43000) [24] (Table S2). Identified SNPs were next reduced to the core SNPs set using the Snippy-core function [40]. Maximum likelihood phylogenetic trees were then constructed with FastTree v2.1.10 [42], using the Shimodaira–Hasegawa (SH) test for local support, and visualized and annotated using R v4.3.1 [43], and the R library ggtree v3.8.2 [44]. Whole-genome alignments were performed and visualized using the progressiveMauve v2.4.0 algorithm [45]. Comparative analyses of multiple genomic regions of interest were conducted using GenoFig v1.1 [46]. Additional R packages with the latest versions were also used for data visualizations [47,48,49].

### 2.5. Screening of Virulence and Regulatory Genes, Lancefield Group Determination, and Antimicrobial Resistance Profiling

To screen for the presence of virulence factors and genes involved in global virulence regulation, we aligned isolate sequence reads to known or potential virulence factors and regulators through the VFanalyzer automatic pipeline at the Virulence Factors of Pathogenic Bacteria Database (VFDB) [50]. BLASTn v2.13.0 [51] was also used to interrogate de novo assemblies for the presence of reported virulence factors and regulators [24,52,53,54]. To determine the Lancefield antigen group of our SDSE strains, we compared their carbohydrate biosynthesis gene clusters with reference clusters representing different Lancefield groups [55] and confirmed serological reactivity using a commercial latex agglutination test kit for groups A, B, C, D, F, and G (Thermo Fisher Scientific). Because the commercial kit does not include group L antisera, strains predicted to belong to this group were classified based on genomic data only. To define isolate AMR gene content, we used ResFinder v4.4.2 [56] and the RGI v6.0.3 from the Comprehensive Antibiotic Resistance Database (CARD) [57]. All queries were executed with default parameters.

### 2.6. Antimicrobial Susceptibility Testing

Antimicrobial susceptibilities were assessed by the Kirby–Bauer disk diffusion method [58] on Mueller–Hinton agar supplemented with 5% defibrinated sheep blood, following Clinical and Laboratory Standards Institute (CLSI) recommendations [59]. The antimicrobial panel included β-lactams (penicillin, 10 units; ceftiofur, 30 µg), macrolide–lincosamide agents (erythromycin, 15 µg; clindamycin, 2 µg), a fluoroquinolone (enrofloxacin, 5 µg), a tetracycline (tetracycline, 30 µg), an aminoglycoside (gentamicin, 10 µg), a phenicol (florfenicol, 30 µg), and trimethoprim–sulfamethoxazole (1.25/23.75 µg). Because CLSI does not provide species-specific interpretive criteria for SDSE, zone diameters were interpreted using breakpoints for *Streptococcus pneumoniae* or *Streptococcus* spp. β-hemolytic group, *Streptococcus suis* or *Streptococcus* spp., or *Staphylococcus* spp., depending on the antimicrobial agent. These surrogate standards represent the most appropriate reference criteria for veterinary diagnostic laboratories. Quality control was performed using *Mannheimia haemolytica* ATCC 33396 and *S. pneumoniae* ATCC 49619. Multidrug-resistant (MDR) and extensively drug-resistant (XDR) classifications were assigned according to internationally accepted definitions, whereby MDR was defined as non-susceptibility to at least one agent in three or more antimicrobial categories and up to (and including) the total number of all antimicrobial categories minus two, and XDR as non-susceptibility to at least one agent in all but two or fewer antimicrobial categories [60,61].

### 2.7. Statistical Analysis and Visualization

Statistical analyses were performed in R v4.3.1 [43]. Fisher’s exact test was used to assess frequency-based differences (e.g., presence or absence of virulence or AMR genes) between strain populations or subpopulations, with *p* < 0.05 considered statistically significant. For analyses implemented within bioinformatics pipelines (e.g., FastTree SH support, ICEscreen clustering, or ResFinder scoring), we used the statistical procedures embedded in the respective software packages.

## 3. Results

### 3.1. Porcine SDSE Isolates Were Recovered from Diverse Age Groups and Disease Presentations, with Increasing Frequency over Time

**Figure 1:**
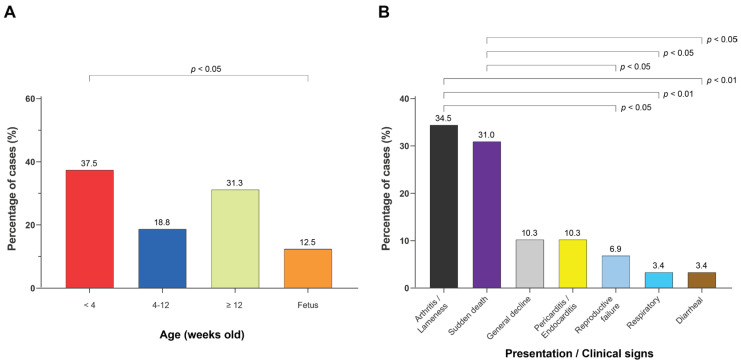
Distribution of *Streptococcus dysgalactiae* subsp. *equisimilis* (SDSE) cases by age group and by presentation/clinical signs. (**A**) Age distribution of SDSE-positive clinical episodes, expressed as the percentage of cases within each category (<4 weeks, 4–12 weeks, ≥12 weeks, and fetus). (**B**) Presentations or clinical signs observed among SDSE-positive cases, including arthritis/lameness, sudden death, general decline, pericarditis/endocarditis, reproductive failure, respiratory, and gastrointestinal involvement. Bars represent the percentage of SDSE-positive cases within each category. Statistical significance of between-group differences was evaluated using the χ^2^ or Fisher’s exact test (*p* < 0.05 or *p* < 0.01, as indicated).

We first examined the host and disease features associated with positive isolation of SDSE. Age information was available for 32 cases, of which 12 (37.5 %) were pigs aged < 4 weeks old, six (18.8 %) pigs aged 4–12 weeks old, and 10 (31.3 %) pigs aged ≥ 12 weeks old. Four cases (12.5%) involved fetuses (Figure 1A). Clinical records were available for 29 cases. The most frequent disease presentations were arthritis and/or lameness (10/29, 34.5%) and sudden death (9/29, 31.0%). Other manifestations included general decline (3/29, 10.3%), pericarditis and/or endocarditis (3/29, 10.3%), reproductive failure (2/29, 6.9%), respiratory signs (1/29, 3.4%), and diarrheal signs (1/29, 3.4%) (Figure 1B). The annual distribution showed an increase over time, with 4/41 (9.8%) collected in 2019, 7/41 (17.1%) in 2020, 9/41 (22.0%) in 2021, and 21/41 (51.2%) in 2022 (Figure S1). Together, these data show that SDSE infection occurred in pigs across multiple production stages and clinical presentations, with fluctuations in case numbers over the study period.

### 3.2. Comparative Genomics Identifies Structural Variation in Porcine SDSE Isolates and Marked Divergence from a Human Reference Strain

**Figure 2:**
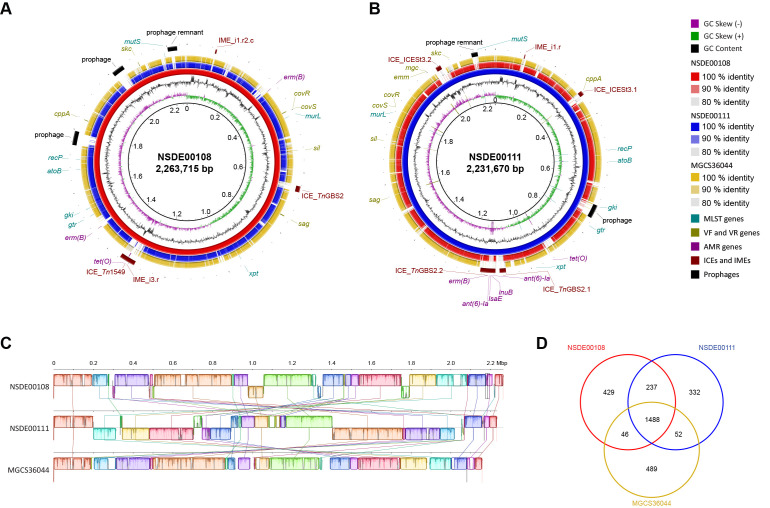
Comparative genomic architecture of *Streptococcus dysgalactiae* subsp. *equisimilis* (SDSE) strains of swine and human origin. (**A**,**B**) Genome atlases of swine-derived SDSE strains NSDE00108 (**A**) and NSDE00111 (**B**). From innermost to outermost circles, rings represent: genome size in Mbp (circle 1); GC skew (G − C)/(G + C) averaged over a 10 kb sliding window, with excess G and excess C indicated in green and purple, respectively (circle 2); GC content (circle 3); in silico TBLASTN comparisons between the swine isolates (circles 4 and 5) and the human invasive strain MGCS36044 (circle 6), with shading intensity corresponding to amino-acid identity (100%, 90%, 80%); and genome landmarks (circle 7) highlighting MLST loci (turquoise), virulence and regulatory (VF/VR) genes (olive), antimicrobial-resistance determinants (magenta), integrative and conjugative or mobilizable elements (ICEs/IMEs; maroon), and prophages (black). (**C**) Pairwise whole-genome alignment between MGCS36044 and the swine strains, generated with progressiveMauve v2.4.0. Colored local collinear blocks (LCBs) represent conserved syntenic regions; inverted blocks denote sequence inversions relative to the reference axis. (**D**) Orthologous-gene comparison among the human and swine SDSE genomes showing total and shared gene-cluster counts derived from Roary analysis. AMR, antimicrobial resistance; VF, virulence factor; VR, virulence regulator; ICE, integrative conjugative element; IME, integrative mobilizable element.

To begin to characterize the porcine SDSE collection of isolates, we first sequenced to closure the genomes of two randomly selected strains using a hybrid strategy combining Oxford Nanopore long-read and Illumina short-read technologies. Figure 2A,B display the genome atlases of these two isolates (NSDE00108 and NSDE00111). The circularized genomes were 2,263,715 and 2,231,670 bp in length, and contained 2200 and 2109 predicted coding sequences, respectively. GC content was 39.55% for both genomes (Table S3).

BLASTN analysis showed a relatively high level of sequence similarity between the porcine isolates, as well as in comparison to the well-characterized human-derived SDSE strain MGCS36044 [41]. However, NSDE00111 contained two large sequence inversions relative to the other strains (Figure 2C). The chromosomes of the porcine isolates also harbored multiple MGEs ranging from 2751 to 49,931 bp, whereas the human strain contained few such elements (Table S4). Comparative gene-content analysis among NSDE00108, NSDE00111, and MGCS36044 showed that the two porcine isolates shared 237 genes not found in the human genome, in contrast to the limited overlap between each porcine strain and MGCS36044 (46 and 52 shared genes, respectively) (Figure 2D). These results indicate greater genomic conservation between porcine SDSE isolates than between pig- and human-derived strains.

### 3.3. Core-Genome Phylogeny and MLST Analysis Reveal Extensive Diversity Among Porcine SDSE and Clear Separation from Human Isolates

**Figure 3:**
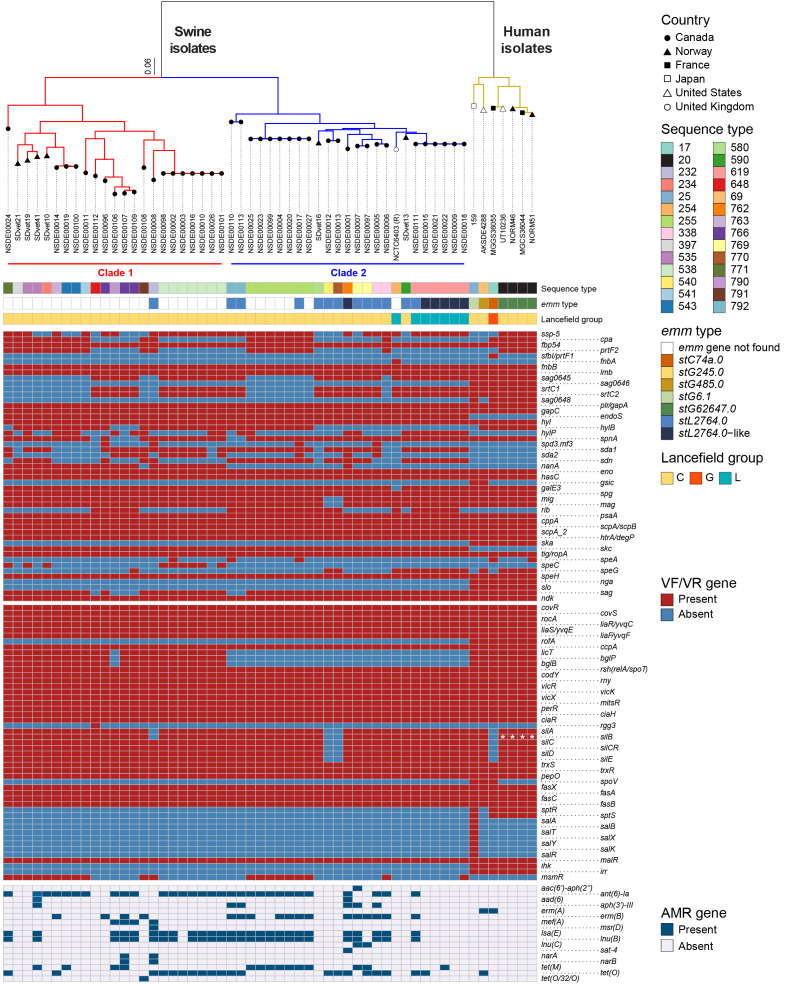
Phylogenomic structure, traditional typing, virulence and regulatory gene profiles, and antimicrobial resistance (AMR) gene content of *Streptococcus dysgalactiae* subsp. *equisimilis* (SDSE) isolates of swine and human origin. A maximum-likelihood phylogeny based on 64,302 core-genome SNPs, identified relative to the genome sequence of the SDSE historic swine strain NCTC6403 (GenBank Accession number: NZ_LR594046) is depicted in the top panel, with swine isolates forming two discrete but heterogeneous clades (left), which are distantly related genetically to human isolates (right). The country of isolation of the strains is indicated by the various open and closed symbols. The scale bar represents substitutions per site. Below the tree, vertical panels summarize sequence type (ST), *emm* type, and Lancefield antigen group (LG). Heatmaps depict the presence (red) or absence (blue) of genes encoding known or putative virulence factors (VF) and virulence regulators (VR). Asterisks in *silB* indicate that the gene is interrupted by a transposon insertion. The last bottom panel shows presence (dark blue) or absence (gray) of antimicrobial resistance genes in the genomes of the isolates.

To characterize the population structure of our isolate collection, we next sequenced the remaining isolates using Illumina short-read technology. We combined these genomes with all publicly available genome data of porcine SDSE, as well as with seven genomes representing the diversity of SDSE isolates associated with human disease. Using this combined dataset, we generated a core-genome alignment and inferred a maximum-likelihood phylogeny (Figure 3). Results showed that porcine SDSE isolates can roughly be grouped into two major clades, each supported by high SH-like local support values and separated by long internal branches. Clade 1 comprised 19 isolates from our collection and four contemporary Norwegian strains, while clade 2 included 22 of our isolates, two contemporary Norwegian strains, and the historic porcine strain NCTC6403. However, the long internal branches within and between these groupings illustrate a relatively extensive genomic diversity of porcine SDSE, consistent with a non-clonal population structure. In contrast, all human isolates cluster tightly within one branch, representing, comparatively, a more homogeneous population (Figure 3).

MLST identified 18 sequence types (STs) among isolates from our collection (Figure 3), including nine novel allele profiles (Table S5). Comparison with the PubMLST database also clarified the ST of two Norwegian isolates (SDvet13 and SDvet16), used here as comparators along with other swine and human isolates, which, in our hands, tested ST590 and ST580 rather than the originally reported [24] ST338 (Table S5). Across all porcine and human SDSE isolates included in the analysis, STs could be grouped into sixteen clonal complexes (CCs) and five singletons. CC538 was the most frequent complex (n = 8), followed by CC620 (n = 7), CC20 (n = 4), CC233 (n = 4), and CC338 (n = 4). Eighteen isolates were distributed across eleven additional CCs, each comprising only one to three isolates. Singletons represented 18.2% of isolates, underscoring extensive allelic diversity. A minimum-spanning tree (MST) constructed from MLST profiles recapitulated the genome-based phylogeny, showing a clear separation between SDSE of swine and human origin (Figure S2). The two host groups differed in all seven loci, with no shared alleles (Table S5).

### 3.4. Virulence and Regulatory Gene Content Differs Between Hosts but Shows No Consistent Pattern Across Porcine Clades

We screened the genomes of all swine and human strains for the presence of 148 known and putative virulence factors. Among swine SDSE isolates from Canada and Norway, 43 factors were identified, increasing to 50 when human isolates were included. Table S6 lists each factor together with the results of homology searches for individual genomes, while Table S7 summarizes aggregated results. Overall, the distribution of virulence factors across clades 1 and 2 showed no consistent lineage-specific pattern (Figure 3). However, seven factors—*hylB* (hyaluronidase), *nanA* (neuraminidase A), *sda2* and *sdn* (streptodornases), *rib* (surface protein Rib), and the superantigen genes *speC* and *speG*—differed significantly in their clade associations. Porcine SDSE genomes uniquely carried eleven virulence genes (*fnbA*, *endoS*, *hylP*, *nanA*, *spd3/mf3*, *sda1*, *sda2*, *sdn*, *rib*, *skc*, and *speC*) that were absent from human SDSE genomes, whereas the latter uniquely possessed *sfbI/prtF1*, two uncharacterized genes from pilus island 1, *gsic*, *ska*, *nga*, and *slo*.

We next screened the genomes of all swine and human SDSE strains for 58 known or putative virulence regulators previously described in SDSE and/or *S. pyogenes*. Table S8 lists these regulators together with their presence or absence in individual genomes. Thirty-six regulators were identified in porcine genomes and up to 53 in human genomes (Table S8). Most regulatory genes were highly conserved across both porcine clades, each showing near-complete presence. Three genes associated with the phosphoenolpyruvate phosphotransferase system (*licT, bglP,* and *bglB*) occurred exclusively in the 22 clade 1 genomes, and the quorum-sensing regulator *rgg3*, a member of the RRNPP family, was likewise confined to clade 1 but detected in only one genome. The aggregate distribution by host species and clade is summarized in Table S9, while the corresponding pattern is depicted in Figure 3. Thirteen regulators were either unique to, or occurred at markedly higher frequency among human genomes, including *rofA* (pilus transcriptional regulator), *spoV* (streptococcal peptide regulator), and the two-component systems *sptR–sptS* and *ihk–irr*.

### 3.5. Loss and Structural Rearrangement of the emm Region Distinguish Porcine SDSE from Human Strains

The *emm* gene encodes the M protein, a major surface virulence factor that mediates adhesion, antiphagocytic activity, and immune evasion in *S. pyogenes* and human-associated SDSE. Given its central role in host interaction, we examined the presence and genomic organization of this locus in porcine isolates. Among the 41 porcine SDSE isolates analyzed, 11 were assigned to *stL2764*, and six carried *stL2764*-like variants with minor sequence mismatches. However, the *emm* gene was absent in 18 of 19 clade 1 and 6 of 23 clade 2 isolates, resulting in *emm*-non-typable genomes (Figure 3).

**Figure 4:**
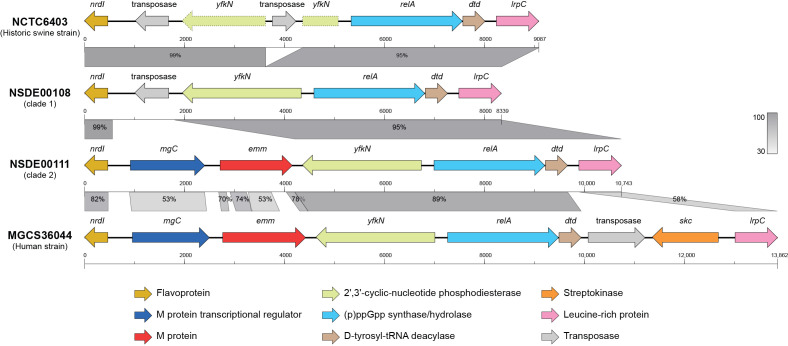
Comparative organization of the *emm* locus region in swine and human *Streptococcus dysgalactiae* subsp. *equisimilis* (SDSE) isolates. Linear comparisons show the chromosomal segment spanning *nrdI* to *lrpC* in the historic swine strain NCTC6403, swine isolates NSDE00108 (clade 1) and NSDE00111 (clade 2), and the human invasive strain MGCS36044. The *emm* locus is absent in NCTC6403 and NSDE00108, present in NSDE00111 and MGCS36044, but a structural difference between the latter two arises from the adjacent *skc* gene, found at that position only in the human strain. The segments with dashed borders in NCTC6403 depict the *yfkN* gene disrupted by insertion of a transposase. Genes are drawn to scale, and percentage values indicate nucleotide identity between homologous regions.

Comparative analysis of the *emm* region—bounded by *nrdI* and *lrpC*—in representative strains revealed marked structural variation (Figure 4). NSDE00108 (a clade 1 isolate) and the historic swine reference strain NCTC6403 shared an identical organization in which a transposase insertion replaced the *emm* locus, while NSDE00111 (a clade 2 isolate) possessed genes *mgC* and *emm* downstream of *nrdI*, displaying an arrangement similar to the human strain MGCS36044. However, there were differences between clade 2 and human strains. In MGCS36044, and in other human SDSE genomes, the gene *skc* (encoding streptokinase C) is located immediately downstream of *emm*, forming part of the canonical *mgC–emm–skc* region. In porcine isolates such as NSDE00111, however, *skc* is located far away from the *emm* locus (Figure 2B). These observations highlight extensive lineage-specific remodeling of the *emm* locus and its surrounding virulence gene neighborhood between porcine and human SDSE.

### 3.6. Variation in Lancefield Antigen Biosynthesis Loci Defines Group C and L Lineages Among Porcine SDSE

**Figure 5:**
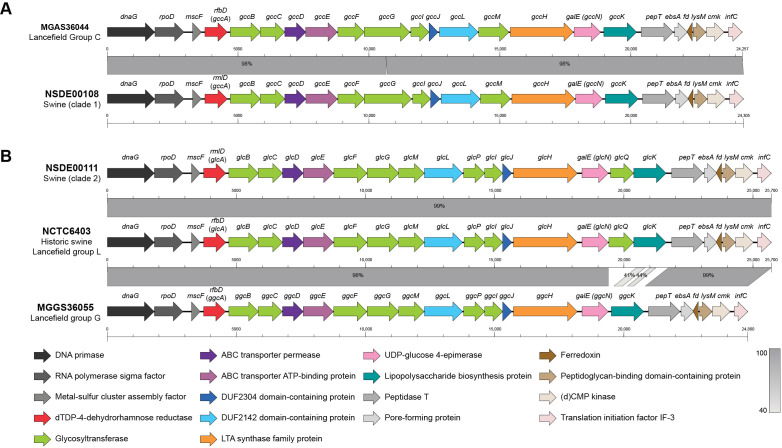
Comparative organization of the Lancefield antigen biosynthesis loci in representative swine-derived *Streptococcus dysgalactiae* subsp. *equisimilis* (SDSE) isolates. (**A**) Alignment of the carbohydrate antigen gene cluster from the human group C reference strain MGCS36044 and a representative swine group C isolate (NSDE00108, clade 1). Both genomes display the conserved locus organization characteristic of Lancefield group C. (**B**) Lancefield antigen biosynthesis locus from a representative porcine group L isolate (NSDE00111, clade 2) compared with those of the historic swine group L strain NCTC6403 and the human group G reference strain MGGS36055. The porcine group L isolates share a similar locus organization, which differs from that of group G in genes *glcK*/*ggcK* (low amino acid similarity) and *glcQ* (only present in group L loci). Genes are drawn to scale and color-coded by predicted function; percent amino acid similarity to the indicated reference genome is indicated by the gray boxes between aligned loci.

The Lancefield antigen, a surface polysaccharide that defines streptococcal serogroups, is synthesized by glycosyltransferases and transport proteins encoded by the *gacA–L* locus in *S. pyogenes* [55]. Phenotypic testing with a commercial latex agglutination kit typed 35 of 41 isolates in our collection as group C, while six others reacted strongly with both group A and G antibodies (Table S1, Figure S3). To better understand the observed phenotypes, we next compared the corresponding carbohydrate synthesis loci of swine SDSE with those of reference strains representing groups C (MGCS36044), G (MGGS36055), and L (NCTC6403). In agreement with phenotypic results, 35 isolates shared high homology across the carbohydrate synthesis gene cluster with the group C strain MGCS36044 (Figure 5A). The remaining six isolates had a gene organization similar to that observed in the Lancefield group L historic swine strain NCTC6403, with all genes of the locus sharing high similarity to those of group G MGGS36055 except for *glcK* and *glcQ* (Figure 5B). Historical serological studies reported that carbohydrate extracts of group L streptococci can cross-react with group A antisera, reflecting shared antigenic determinants between these groups [62,63]. Combined with the close genomic similarity between group L and group G loci, these data provide a plausible explanation for the strong A/G latex agglutination observed in these six isolates. Thus, despite the absence of a group L reaction in the commercial kit we used, based on genomic organization, we determined that these six isolates belong to Lancefield group L. No group G SDSE isolates were identified in our collection.

### 3.7. Widespread Antimicrobial Resistance and Multidrug-Resistant Phenotypes Among Porcine SDSE

The 41 swine SDSE isolates were tested for antimicrobial susceptibility using the Kirby–Bauer disk diffusion method and a standard veterinary antibiotic panel. All isolates were susceptible to β-lactam agents, including penicillin and ceftiofur, as well as to florfenicol (Table 1). High susceptibility was also observed for enrofloxacin (87.8%) and gentamicin (90.2%), while most isolates remained sensitive to trimethoprim–sulfamethoxazole (75.6%) (Table 1). In contrast, reduced susceptibility was frequent among macrolides, with 61.0% of isolates susceptible to erythromycin, whereas resistance was common for clindamycin (68.3%) and high for tetracycline (82.9%) (Table 1).

We next investigated the AMR gene content of our isolates and found 17 AMR genes. The most frequently detected aminoglycoside resistance gene was *ant(6)-Ia* (68.3%) (Figure 3; Table S10), which encodes an enzyme active against streptomycin but not gentamicin. Additional aminoglycoside-modifying enzymes were less common: *aph(3′)-III* was detected in five isolates (12.2%), *aad(6)* in one, and one isolate harbored the bifunctional *aac(6′)-aph(2″)* gene together with *aph(3′)-III*. Only one gentamicin-resistant isolate carried canonical gentamicin resistance determinants (*aac(6′)-aph(2″)* plus *aph(3′)-III*), whereas the second resistant and the two intermediate isolates lacked known gentamicin-specific resistance genes. This partial discordance likely reflects the variability known to affect gentamicin disk diffusion testing in streptococci [59,64].

Genes *tet(M)*, *tet(O)* and/or *tet(O/32/O)* were detected in all SDSE isolates resistant to tetracycline (Figure 3, Table S10). Other frequently observed genes included *erm(B)* (41.5%), which confers macrolide–lincosamide–streptogramin B (MLSB) resistance; *lsa(E)* (61.0%), associated with resistance to lincosamides, pleuromutilins, and streptogramins; as well as *lnu(B)* (61.0%) and *lnu(C)* (4.9%), both linked to resistance against lincosamides. In most cases, the presence of macrolide- and lincosamide-resistance determinants correlated well with phenotypic susceptibility profiles (Table S10). However, a small number of discordant profiles were identified. Two *erm(B)*-positive and two *mef(A)*-positive isolates were susceptible to erythromycin. Likewise, one isolate possessing *lsa(E)* and *lnu(B)* showed a clindamycin-susceptible phenotype.

Consistent with uniform florfenicol susceptibility observed in vitro, no transferable phenicol resistance genes (e.g., *optrA*, *fexA*, *poxtA*) were identified. Furthermore, we found no specific point mutation associated with phenotypic resistance within fluoroquinolone resistance genes, and targeted inspection of *gyrA*/*parC* in a subset of intermediate susceptible isolates revealed no canonical QRDR substitutions. Similarly, no acquired trimethoprim (*dfr*) or sulfonamide (*sul*) resistance genes were identified, consistent with the high susceptibility to trimethoprim–sulfamethoxazole observed in vitro. The few non-susceptible isolates likely reflect natural variation in folate-pathway susceptibility that does not correspond to known acquired determinants.

When we examined multidrug-resistance (MDR) profiles, we identified thirteen distinct MDR patterns among porcine isolates. Based on current definitions for multidrug-resistant (MDR) and extensively drug-resistant (XDR) bacteria [60,61], 14 isolates (34.1%) were classified as MDR and 15 (36.6%) as XDR (Table 2). The most frequent resistance profile, observed in 12 isolates, encompassed six antimicrobial classes: aminoglycosides, macrolides, streptogramins, lincosamides, pleuromutilins, and tetracyclines.

## 4. Discussion

### 4.1. Occurrence and Establishment of SDSE in Swine

Although SDSE is best known as an opportunistic human pathogen responsible for pharyngitis, cellulitis, and invasive bacteremia [7,24,65], the organism has also been associated with disease in pigs for far longer than is often acknowledged. Indeed, β-hemolytic streptococci consistent with SDSE have been isolated from diseased pigs since at least the 1930–1940s, with early surveys by Hare et al. [66] identifying Lancefield groups C and L streptococci in outbreaks of septicemia, arthritis, and endocarditis in swine herds in England. Subsequent work throughout the mid-20th century showed that group C was the dominant serogroup in pigs, with group L forming a smaller but recurrent minority [67,68].

These early investigations, conducted well before SDSE was formally defined, documented an ecological pattern very similar to the one we report here. Contemporary work from Asia and Europe echoes our data, with SDSE increasingly identified in diseased swine [16,17,18,19,20,22]. Supporting continuous endemic SDSE circulation in Canadian swine herds, our results show that the organism can affect pigs spanning different production stages, resulting in multiple clinical presentations. Across different contemporary studies, SDSE has been detected both in clinical submissions (e.g., Korea, Italy, Sweden) and in slaughterhouse surveillance (e.g., Brazil, India), with invasive lesions frequently described in clinical cases [16,17,18,22,69,70]. In Korea, SDSE was isolated from piglets presenting with lameness, arthritis, and neurological signs [16,17]. In Norway, Porcellato et al. [24] included several pig-derived SDSE genomes within a multi-host dataset. In India, Patel et al. [22] examined 627 slaughtered pigs and recovered 177 SDSE isolates, documenting systemic infection with septicemia and endocarditis. Together with reports from clinically healthy or subclinical animals at slaughter in Brazil [18], these observations verify that SDSE is an established component of the porcine microbiological landscape whose clinical relevance is only now being fully appreciated, giving the appearance of an emerging swine pathogen despite its long-standing presence.

### 4.2. Clinical Features of Porcine SDSE: Contemporary Patterns and Historical Parallels

Isolates from our collection were most frequently recovered from pigs with systemic disease, including arthritis, endocarditis, and sudden death. Cases occurred across multiple production stages, from nursery to grower–finisher animals, indicating that SDSE infections are not restricted to a specific age group. Comparable disease presentations have been described internationally. In Korea, SDSE was isolated from piglets exhibiting lameness, neurological signs, and fibrinous pleuritis or pericarditis, frequently accompanied by high morbidity and mortality [16,17], and work in piglets links lameness to skin/umbilical lesions and abrasions as portals of entry [71]. In Sweden, the organism was recovered mainly from finishing pigs with polyserositis or purulent arthritis [69], whereas in Italy and India, isolates were from animals with septicemia, endocarditis, or chronic joint infections consistent with persistent bacteremia [22,70]. These geographically diverse reports converge on a reproducible clinical profile dominated by invasive, multisystemic infection rather than localized or mucosal disease. Although detailed gross or histopathological lesion descriptions were not uniformly available for all retrospective diagnostic submissions in our study, the observed clinical presentations and isolation sites (Table S1) similarly indicate a predominance of invasive disease, consistent with these international reports. Overall, the available evidence indicates that SDSE infection in pigs consistently targets joints, serosal membranes, and the cardiovascular system, a pathologic spectrum that, while overlapping in part, differs in emphasis from human SDSE disease, which most often presents with bacteremia, soft-tissue, and osteoarticular infections [7].

SDSE clinical manifestations in pigs reported here and in other contemporary studies are nearly identical to those reported in 1942 by Hare et al. [66]. Furthermore, the gross and microscopic lesions reported in diagnostic submissions, e.g., fibrinous arthritis, valvular endocarditis, and polyserositis, also align with other earlier descriptions of SDSE infections [72]. The recurrence of the same disease spectrum today suggests that porcine SDSE has been a persistent but largely neglected component of swine pathology for decades. This long-standing but under-recognized disease pattern raises the question of why SDSE did not remain firmly established in the swine pathology literature. We speculate that the emergence of *S. suis* as a major porcine pathogen in the 1980s likely shifted diagnostic and research attention away from these group C and L streptococci, contributing to the impression that they were rare or incidental.

Most SDSE isolates in our collection were obtained from necropsies of animals showing gross lesions consistent with systemic infection, thus providing strong presumptive evidence of etiological involvement. However, bacteriological recovery from a normally sterile site does not, by itself, prove disease causation, as contamination or post-mortem translocation cannot be fully excluded. Definitive confirmation of SDSE pathogenicity would require controlled experimental infections.

### 4.3. Extensive Genomic Diversity Among Porcine SDSE and Divergence from Human-Derived Strains

Our genomic analysis resolved two well-supported major porcine clades separated by long internal branches, likely indicating independent diversification. Within each clade, however, isolates displayed a relatively high level of genetic diversity, forming a heterogeneous, non-clonal population. Isolates from different herds and production stages were interspersed across both clades, indicating no clear phylogeographic or epidemiological structuring. Comparable genetic diversity has been reported in Korea and Europe, where multiple SDSE genetic backgrounds were recovered from pigs, sometimes within the same herd [16,17,24].

Historical surveys reported that group C streptococci were the dominant serogroup in diseased pigs, with group L occurring at lower frequency and group G nearly absent [66,67,68,72]. The phylogenetic structure observed in our study aligns only partially with these classical serological observations. Clade 1 consists exclusively of group C isolates, whereas clade 2 contains a mixture of group C and group L isolates. Group L isolates nevertheless represent only a subset of the broader clade 2 diversity and are, interestingly, more closely related genetically to the historic isolate NCTC6403 recovered in the United Kingdom in the 1940s. The repeated recovery of group C and group L isolates across historical and modern datasets suggests that serogroup diversity within porcine SDSE has remained stable for decades.

When compared with human SDSE, the porcine lineages formed clusters distinct from the dominant human clonal complexes CC20, CC17, and CC29, which account for most invasive disease in Europe [7,41,65]. Analyses of human SDSE populations have revealed extensive recombination, particularly within the *emm* locus and other surface-associated genes, and the emergence of the globally disseminated *stG62647*/CC20 complex [41]. In contrast, our porcine SDSE isolates were typically *emm*-negative or contained divergent *emm*-region structures, consistent with previous findings in Asian and European pig isolates [16,24]. Together, these observations indicate that human and porcine SDSE represent largely distinct evolutionary lineages that circulate in different host environments, reflecting host-specific adaptation. This phylogenetic separation may be paralleled by differences in virulence arsenals. For example, several virulence factors were associated with either swine or human clades. In addition, although swine SDSE genomes encode many of the same virulence determinants and global regulators described in human SDSE and *S. pyogenes* (e.g., *sagA*, *scpA*, and the *covRS* regulators), functional equivalence between hosts cannot be assumed. The differing disease manifestations in pigs suggest that these homologues may not fulfill identical roles in the porcine host, or that their expression and regulatory hierarchies may have diverged over time. Experimental validation of these hypotheses will require targeted functional studies.

### 4.4. Antimicrobial Resistance Reservoir Potential of Swine SDSE

All 41 SDSE isolates characterized here were susceptible to β-lactams. Reports from Korea and Brazil similarly describe preserved susceptibility to penicillin and related β-lactams among porcine SDSE isolates [16,17,18]. Interestingly, a recent study from India [22] reported 100% resistance to multiple β-lactams, including penicillin, oxacillin, and even meropenem among tested swine SDSE isolates. These findings differ markedly from established susceptibility profiles for SDSE and therefore warrant cautious interpretation pending independent confirmation. We did not detect any florfenicol resistance, although two *optrA*-positive porcine SDSE isolates have been described in Italy [70]. Additionally, we did not identify enrofloxacin-resistant isolates, although five showed intermediate susceptibility to enrofloxacin by disk diffusion. No canonical QRDR substitutions were identified in *parC* or *gyrA* among these isolates, a finding that contrasts with a previous report from Korea in which fluoroquinolone non-susceptibility was associated with mutations in these loci [16].

On the other hand, our isolates had multiple acquired AMR determinants, including *tet(M)*, *tet(O)*, *erm(B)*, *lnu(B)*, and *lsa(E)*, reflecting a diverse resistome and frequent MDR. Genotype–phenotype concordance was high for most antimicrobial classes, although a small number of macrolide–lincosamide mismatches were identified, which most plausibly reflect non-expression or low expression of certain resistance determinants under the test conditions rather than true genotype–phenotype discordance. For aminoglycosides, the presence of streptomycin-resistance genes (e.g., *ant(6)-Ia*) was common, whereas gentamicin resistance was rare and only partially explained by known aminoglycoside-modifying enzymes, consistent with the known variability of gentamicin disk diffusion in β-hemolytic streptococci. Our isolates were recovered from diseased animals that were likely exposed to antimicrobials prior to necropsy. However, comparable resistance repertoires have been documented internationally. In Japan and Korea, most porcine SDSE isolates carried *tet(M), tet(O)* and/or *erm(B)* [16,20]. Although these studies only performed phenotypic testing, Indian and Brazilian SDSE isolates from slaughtered pigs had broad multidrug-resistance phenotypes [22,73]. By comparison, human SDSE populations display more variable AMR levels that remain generally low in Northern Europe, where macrolide and tetracycline resistance are less common [74], but considerably higher in parts of Asia. For example, surveillance studies in China reported erythromycin and clindamycin resistance rates exceeding 70%, and tetracycline resistance around 60% among clinical SDSE isolates [75], while Japanese datasets documented rapid expansion of macrolide resistance, often carried by ICEs and IMEs [76].

At present, there is no definitive evidence that swine isolates are transmitted to close-contact workers in the pig industry. However, in our dataset, the two circularized genomes carried AMR genes embedded within ICEs and IMEs, providing a plausible framework for horizontal transfer of resistance determinants within and between streptococcal species. Although demonstration of transmissibility will require experimental validation, it is tempting to speculate that, similar to other swine-associated bacteria [77,78], porcine SDSE may contribute to the broader circulation of resistance genes within the farm microbiome.

## 5. Conclusions

We present here a population-scale genomic characterization of SDSE isolates from diseased pigs in Quebec, Canada. By integrating epidemiologic, genomic, and phenotypic data, we show that SDSE circulates endemically across production stages and represents a genetically diverse, host-adapted population distinct from human-derived lineages. The loss or structural remodeling of the *emm* region, variation in Lancefield antigen biosynthesis loci defining groups C and L, and differences in virulence and regulatory gene repertoires all point to long-term evolution within the swine host. Phenotypic analyses confirmed the expression of major genotypic resistance determinants, and complete genome assemblies revealed MGEs that may mediate horizontal AMR gene exchange. Together with historic reports and the growing body of international literature, our findings establish that swine SDSE represents a longstanding, host-adapted population distinct from human strains that may act as an AMR reservoir. Continued monitoring, expanded genomic sampling, and targeted functional studies are needed to clarify its pathogenic mechanisms, ecological persistence, and potential zoonotic implications.

## Figures and Tables

**Table 1:** Kirby-Bauer disk diffusion test of porcine SDSE isolates.

Antimicrobial	Category ^d^	Zone Diameter Breakpoints (mm)			Number of Isolates		
		Susceptible	Intermediate	Resistant	Susceptible (n, %)	Intermediate (n, %)	Resistant (n, %)
Penicillin ^a^	II	≥24	–	–	41 (100)	0 (0)	0 (0)
Ceftiofur ^b^	I	≥21	20–18	≤17	41 (100)	0 (0)	0 (0)
Florfenicol ^b^	III	≥22	21–19	≤18	41 (100)	0 (0)	0 (0)
Enrofloxacin ^b^	I	≥23	22–17	≤16	36 (87.8)	5 (12.2)	0 (0)
Gentamicin ^c^	II	≥15	14–13	≤12	37 (90.2)	2 (4.9)	2 (4.9)
Trimethoprim- sulfamethoxazole ^a^	II	≥19	18–16	≤15	31 (75.6)	8 (19.5)	2 (4.9)
Erythromycin ^a^	II	≥21	20–16	≤15	25 (61.0)	0 (0)	16 (39.0)
Clindamycin ^a^	II	≥19	18–16	≤15	13 (31.7)	0 (0)	28 ^e^ (68.3)
Tetracycline ^a^	III	≥23	22–19	≤18	0 (0)	7 (17.1)	34 (82.9)

^a^ Breakpoints for *S. pneumoniae* or β-hemolytic *Streptococcus* spp., CLSI M100 35th edition [64]. ^b^ Breakpoints for *S. suis* or *Streptococcus* spp., CLSI VET01 6th edition [59]. ^c^ Breakpoints for *Staphylococcus* spp., CLSI M100 35th edition [64]. ^d^ Categorization of antimicrobial drugs based on their importance in human medicine according to Health Canada (Veterinary Drugs Directorate), version April 2009; see https://www.canada.ca/en/health-canada/services/drugs-health-products/veterinary-drugs/antimicrobial-resistance/categorization-antimicrobial-drugs-based-importance-human-medicine.html (accessed on 1 November 2025). Category I: very high importance; Category II: high importance; Category III: medium importance; Category IV: low importance. Note: other international classification frameworks exist, including the World Health Organization List of Medically Important Antimicrobials, which may differ from the Health Canada categorization. ^e^ Sixteen isolates were resistant to erythromycin and showed constitutive MLSB phenotype in the D-test, while the remaining clindamycin-resistant isolates were susceptible to erythromycin.

**Table 2:** Presumptive antimicrobial resistance patterns identified among the 41 porcine SDSE strains included in this study.

Number of Drug Class Combinations	Antimicrobial Resistance Pattern	Number of Isolates (%)
0	None	4 (9.8)
1	Tetracycline	4 (9.8)
	Aminoglycoside	2 (4.9)
2	Aminoglycoside-Tetracycline	2 (4.9)
3 ^a^	Macrolide-Streptogramin-Lincosamide	1 (2.4)
4 ^a^	Macrolide-Streptogramin-Lincosamide-Tetracycline	2 (4.9)
5 ^a^	Aminoglycoside-Streptogramin-Lincosamide-Pleuromutilin-Tetracycline	9 (22.0)
5 ^a^	Aminoglycoside-Macrolide-Streptogramin-Lincosamide-Tetracycline	1 (2.4)
5 ^a^	Aminoglycoside-Macrolide-Streptogramin-Lincosamide-Pleuromutilin	1 (2.4)
6 ^b^	Aminoglycoside-Macrolide-Streptogramin-Lincosamide-Pleuromutilin-Tetracycline	12 (29.3)
6 ^b^	Macrolide-Streptogramin-Lincosamide-Pleuromutilin-Tetracycline-Ionophores	1 (2.4)
7 ^b^	Aminoglycoside-Macrolide-Streptogramin-Lincosamide-Pleuromutilin-Nucleoside-Tetracycline	1 (2.4)
7 ^b^	Aminoglycoside-Macrolide-Streptogramin-Lincosamide-Pleuromutilin-Tetracycline-Ionophores	1 (2.4)

^a^ MDR: multidrug-resistant, which is defined as non-susceptibility to at least one agent in three or more antimicrobial categories and up to (and including) the total number of all antimicrobial categories minus two. ^b^ XDR: extensively drug-resistant, which is defined as non-susceptibility to at least one agent in all but two or fewer antimicrobial categories (i.e., bacterial isolates remain susceptible to only one or two or even none antimicrobial category [in this latter setting bacterial isolates are resistant to at least one antimicrobial agent in all antimicrobial categories and concurrently there is at least one antimicrobial agent to which the isolate is susceptible to]) [60,61].

## Data Availability

Genome data newly generated in this study were deposited in NCBI Sequence Read Archive under BioProject (Accession ID: PRJNA1099248). Individual isolate genome accession numbers can be found in Table S1 of the Supplementary Materials. Novel MLST allelic profiles have been submitted to pubMLST.

## Supplementary Materials

The following supporting information can be downloaded at https://www.mdpi.com/article/10.3390/microorganisms14010009/s1, Figure S1: Annual distribution of *Streptococcus dysgalactiae* subsp. *equisimilis* (SDSE) cases identified in swine submissions from 2019 to 2022; Figure S2: Minimum-spanning tree (MST) based on multilocus sequence typing (MLST) allelic profiles of *Streptococcus dysgalactiae* subsp. *equisimilis* (SDSE) isolates from swine and human infections; Figure S3: Lancefield grouping of two representative porcine *Streptococcus dysgalactiae* subsp. *equisimilis* (SDSE) isolates using the Oxoid latex agglutination kit; Tables S1: Characteristics of the 41 swine SDSE isolates used in this study; Table S2: Additional SDSE genomes included in this study; Table S3: Genome features of porcine isolates NSDE00108 and NSDE00111 and human strain MGCS36044; Table S4: Mobile genetic elements (MGEs) identified in porcine isolates NSDE00108 and NSDE00111 and human strain MGCS36044; Table S5: Multilocus sequence typing (MLST) allelic profiles, sequence types (STs) and clonal complexes (CCs) of SDSE genomes included in this study; Table S6: Screening of 148 virulence-associated genes among porcine and human SDSE genomes; Table S7: Prevalence of virulence factors among porcine and human SDSE genomes included in this study; Table S8: Screening of 58 virulence regulators genes among porcine and human SDSE genomes; Table S9: Prevalence of virulence regulators among porcine and human SDSE genomes included in this study; Table S10: Antimicrobial resistance gene content in SDSE strains from pigs and humans, and results of Kirby-Bauer antimicrobial susceptibility testing.

## Abbreviations

The following abbreviations are used in this manuscript:

AMR	Antimicrobial resistance
CARD	Comprehensive Antibiotic Resistance Database
CC	Clonal complex
CDVUM	Centre de diagnostic vétérinaire de l’Université de Montréal
CLSI	Clinical and Laboratory Standards Institute
CovRS	Control of virulence regulon
ICE	Integrative and conjugative element
IME	Integrative mobilizable element
LCB	Local collinear block
MALDI-TOF MS	Matrix-assisted laser desorption/ionization time-of-flight mass spectrometry
MAPAQ	Ministère de l’Agriculture, des Pêcheries et de l’Alimentation du Québec
MDR	Multidrug-resistant
MGE	Mobile genetic element
MLST	Multilocus sequencing typing
MST	Minimum-spanning tree
ONT	Oxford Nanopore Technologies
SDSE	Streptococcus *dysgalactiae* subsp. *equisimilis*
Sil	Streptococcal invasive locus
SLV	Single-locus variant
SNP	Single-nucleotide polymorphism
ST	Sequence type
VF	Virulence factor
VFDB	Virulence Factors of Pathogenic Bacteria Database
VR	Virulence regulator
XDR	Extensively drug-resistant

## References

[1] Brandt C.M., Spellerberg B. (2009). Human infections due to *Streptococcus dysgalactiae* subspecies *equisimilis*. Clin. Infect. Dis..

[2] Vandamme P., Pot B., Falsen E., Kersters K., Devriese L.A. (1996). Taxonomic study of lancefield streptococcal groups C, G, and L (*Streptococcus dysgalactiae*) and proposal of *S. dysgalactiae* subsp. *equisimilis* subsp. nov. Int. J. Syst. Bacteriol..

[3] Facklam R. (2002). What happened to the streptococci: Overview of taxonomic and nomenclature changes. Clin. Microbiol. Rev..

[4] Chochua S., Rivers J., Mathis S., Li Z., Velusamy S., McGee L., Van Beneden C., Li Y., Metcalf B.J., Beall B. (2019). Emergent Invasive Group A *Streptococcus dysgalactiae* subsp. *equisimilis*, United States, 2015–2018. Emerg. Infect. Dis..

[5] Takahashi T., Ubukata K., Watanabe H. (2011). Invasive infection caused by *Streptococcus dysgalactiae* subsp. *equisimilis*: Characteristics of strains and clinical features. J. Infect. Chemother..

[6] Sunaoshi K., Murayama S.Y., Adachi K., Yagoshi M., Okuzumi K., Chiba N., Morozumi M., Ubukata K. (2010). Molecular *emm* genotyping and antibiotic susceptibility of *Streptococcus dysgalactiae* subsp. *equisimilis* isolated from invasive and non-invasive infections. J. Med. Microbiol..

[7] Oppegaard O., Mylvaganam H., Skrede S., Lindemann P.C., Kittang B.R. (2017). Emergence of a *Streptococcus dysgalactiae* subspecies *equisimilis stG62647*-lineage associated with severe clinical manifestations. Sci. Rep..

[8] Leitner E., Zollner-Schwetz I., Zarfel G., Masoud-Landgraf L., Gehrer M., Wagner-Eibel U., Grisold A.J., Feierl G. (2015). Prevalence of *emm* types and antimicrobial susceptibility of *Streptococcus dysgalactiae* subsp. *equisimilis* in Austria. Int. J. Med. Microbiol..

[9] Bruun T., Kittang B.R., de Hoog B.J., Aardal S., Flaatten H.K., Langeland N., Mylvaganam H., Vindenes H.A., Skrede S. (2013). Necrotizing soft tissue infections caused by *Streptococcus pyogenes* and *Streptococcus dysgalactiae* subsp. *equisimilis* of groups C and G in western Norway. Clin. Microbiol. Infect..

[10] Xie O., Davies M.R., Tong S.Y.C. (2024). *Streptococcus dysgalactiae* subsp. *equisimilis* infection and its intersection with *Streptococcus pyogenes*. Clin. Microbiol. Rev..

[11] Watanabe S., Takemoto N., Ogura K., Miyoshi-Akiyama T. (2016). Severe invasive streptococcal infection by *Streptococcus pyogenes* and *Streptococcus dysgalactiae* subsp. *equisimilis*. Microbiol. Immunol..

[12] Bustos C.P., Retamar G., Leiva R., Frosth S., Ivanissevich A., Demarchi M.E., Walsh S., Frykberg L., Guss B., Mesplet M. (2023). Novel Genotype of *Streptococcus dysgalactiae* subsp. *equisimilis* Associated with Mastitis in an Arabian Filly: Genomic Approaches and Phenotypic Properties. J. Equine Vet. Sci..

[13] Pinho M.D., Erol E., Ribeiro-Goncalves B., Mendes C.I., Carrico J.A., Matos S.C., Preziuso S., Luebke-Becker A., Wieler L.H., Melo-Cristino J. (2016). Beta-hemolytic *Streptococcus dysgalactiae* strains isolated from horses are a genetically distinct population within the *Streptococcus dysgalactiae* taxon. Sci. Rep..

[14] Silva L.G., Genteluci G.L., Correa de Mattos M., Glatthardt T., Sa Figueiredo A.M., Ferreira-Carvalho B.T. (2015). Group C *Streptococcus dysgalactiae* subsp. *equisimilis* in south-east Brazil: Genetic diversity, resistance profile and the first report of human and equine isolates belonging to the same multilocus sequence typing lineage. J. Med. Microbiol..

[15] Gottschalk M., Segura M., de Oliveira Costa M. (2025). *Streptococcus* spp.. Diseases of Swine.

[16] Oh S.I., Kim J.W., Kim J., So B., Kim B., Kim H.Y. (2020). Molecular subtyping and antimicrobial susceptibility of *Streptococcus dysgalactiae* subspecies *equisimilis* isolates from clinically diseased pigs. J. Vet. Sci..

[17] Oh S.I., Kim J.W., Jung J.Y., Chae M., Lee Y.R., Kim J.H., So B., Kim H.Y. (2018). Pathologic and molecular characterization of *Streptococcus dysgalactiae* subsp. *equisimilis* infection in neonatal piglets. J. Vet. Sci..

[18] Moreno L.Z., da Costa B.L., Matajira C.E., Gomes V.T., Mesquita R.E., Silva A.P., Moreno A.M. (2016). Molecular and antimicrobial susceptibility profiling of *Streptococcus dysgalactiae* isolated from swine. Diagn. Microbiol. Infect. Dis..

[19] Kasuya K., Yoshida E., Harada R., Hasegawa M., Osaka H., Kato M., Shibahara T. (2014). Systemic *Streptococcus dysgalactiae* subspecies *equisimilis* infection in a Yorkshire pig with severe disseminated suppurative meningoencephalomyelitis. J. Vet. Med. Sci..

[20] Fujimoto H., Tanaka T., Nishiya H., Gunji Y., Uto S., Inoue M., Chuma T. (2013). Antimicrobial Susceptibilities and Resistant Genes in β-hemolytic *Streptococci* Isolated from Endocarditis in Slaughtered Pigs. J. Jpn. Vet. Med. Assoc..

[21] Kawata K., Minakami T., Mori Y., Katsumi M., Kataoka Y., Ezawa A., Kikuchi N., Takahashi T. (2003). rDNA sequence analyses of *Streptococcus dysgalactiae* subsp. *equisimilis* isolates from pigs. Int. J. Syst. Evol. Microbiol..

[22] Patel S.M., Sahoo M., Thakor J.C., Murali D., Kumar P., Singh R., Singh K.P., Saikumar G., Jana C., Patel S.K. (2024). Pathomolecular epidemiology, antimicrobial resistance, and virulence genes of *Streptococcus dysgalactiae* subsp. *equisimilis* isolates from slaughtered pigs in India. J. Appl. Microbiol..

[23] Schrieber L., Towers R., Muscatello G., Speare R. (2014). Transmission of *Streptococcus dysgalactiae* subsp. *equisimilis* between child and dog in an Aboriginal Australian community. Zoonoses Public Health.

[24] Porcellato D., Smistad M., Skeie S.B., Jorgensen H.J., Austbo L., Oppegaard O. (2021). Whole genome sequencing reveals possible host species adaptation of *Streptococcus dysgalactiae*. Sci. Rep..

[25] Sayers E.W., Beck J., Bolton E.E., Brister J.R., Chan J., Connor R., Feldgarden M., Fine A.M., Funk K., Hoffman J. (2025). Database resources of the National Center for Biotechnology Information in 2025. Nucleic Acids Res.

[26] Li K., Lacouture S., Lewandowski E., Thibault E., Gantelet H., Gottschalk M., Fittipaldi N. (2024). Molecular characterization of *Streptococcus suis* isolates recovered from diseased pigs in Europe. Vet. Res..

[27] Wood D.E., Lu J., Langmead B. (2019). Improved metagenomic analysis with Kraken 2. Genome Biol..

[28] Inouye M., Dashnow H., Raven L.-A., Schultz M.B., Pope B.J., Tomita T., Zobel J., Holt K.E. (2014). SRST2: Rapid genomic surveillance for public health and hospital microbiology labs. Genome Med..

[29] Jolley K.A., Bray J.E., Maiden M.C.J. (2018). Open-access bacterial population genomics: BIGSdb software, the PubMLST.org website and their applications. Wellcome Open Res..

[30] McMillan D.J., Bessen D.E., Pinho M., Ford C., Hall G.S., Melo-Cristino J., Ramirez M. (2010). Population genetics of *Streptococcus dysgalactiae* subspecies *equisimilis* reveals widely dispersed clones and extensive recombination. PLoS ONE.

[31] Nascimento M., Sousa A., Ramirez M., Francisco A.P., Carrico J.A., Vaz C. (2017). PHYLOViZ 2.0: Providing scalable data integration and visualization for multiple phylogenetic inference methods. Bioinformatics.

[32] Athey T.B., Teatero S., Li A., Marchand-Austin A., Beall B.W., Fittipaldi N. (2014). Deriving group A *Streptococcus* typing information from short-read whole-genome sequencing data. J. Clin. Microbiol..

[33] Wick R.R., Judd L.M., Gorrie C.L., Holt K.E. (2017). Unicycler: Resolving bacterial genome assemblies from short and long sequencing reads. PLoS Comput. Biol..

[34] Seemann T. (2014). Prokka: Rapid prokaryotic genome annotation. Bioinformatics.

[35] Page A.J., Cummins C.A., Hunt M., Wong V.K., Reuter S., Holden M.T., Fookes M., Falush D., Keane J.A., Parkhill J. (2015). Roary: Rapid large-scale prokaryote pan genome analysis. Bioinformatics.

[36] Johansson M.H.K., Bortolaia V., Tansirichaiya S., Aarestrup F.M., Roberts A.P., Petersen T.N. (2021). Detection of mobile genetic elements associated with antibiotic resistance in *Salmonella enterica* using a newly developed web tool: MobileElementFinder. J. Antimicrob. Chemother..

[37] Lao J., Lacroix T., Guedon G., Coluzzi C., Payot S., Leblond-Bourget N., Chiapello H. (2022). ICEscreen: A tool to detect Firmicute ICEs and IMEs, isolated or enclosed in composite structures. NAR Genom. Bioinform..

[38] Wishart D.S., Han S., Saha S., Oler E., Peters H., Grant J.R., Stothard P., Gautam V. (2023). PHASTEST: Faster than PHASTER, better than PHAST. Nucleic Acids Res..

[39] Tritt A., Eisen J.A., Facciotti M.T., Darling A.E. (2012). An integrated pipeline for de novo assembly of microbial genomes. PLoS ONE.

[40] Seemann T. Snippy: Rapid Haploid Variant Calling and Core Genome Alignment. GitHub. 2020. https://github.com/tseemann/snippy.

[41] Beres S.B., Olsen R.J., Long S.W., Eraso J.M., Boukthir S., Faili A., Kayal S., Musser J.M. (2023). Analysis of the Genomics and Mouse Virulence of an Emergent Clone of *Streptococcus dysgalactiae* Subspecies *equisimilis*. Microbiol. Spectr..

[42] Price M.N., Dehal P.S., Arkin A.P. (2010). FastTree 2—Approximately maximum-likelihood trees for large alignments. PLoS ONE.

[43] R Core Team (2023). R: A Language and Environment for Statistical Computing.

[44] Xu S., Li L., Luo X., Chen M., Tang W., Zhan L., Dai Z., Lam T.T., Guan Y., Yu G. (2022). Ggtree: A serialized data object for visualization of a phylogenetic tree and annotation data. Imeta.

[45] Darling A.E., Mau B., Perna N.T. (2010). progressiveMauve: Multiple genome alignment with gene gain, loss and rearrangement. PLoS ONE.

[46] Branger M., Leclercq S.O. (2024). GenoFig: A user-friendly application for the visualization and comparison of genomic regions. Bioinformatics.

[47] Gu Z., Eils R., Schlesner M. (2016). Complex heatmaps reveal patterns and correlations in multidimensional genomic data. Bioinformatics.

[48] Gao C.H., Yu G., Cai P. (2021). ggVennDiagram: An Intuitive, Easy-to-Use, and Highly Customizable R Package to Generate Venn Diagram. Front. Genet..

[49] Gu Z. (2022). Complex heatmap visualization. Imeta.

[50] Liu B., Zheng D., Zhou S., Chen L., Yang J. (2022). VFDB 2022: A general classification scheme for bacterial virulence factors. Nucleic Acids Res..

[51] Altschul S.F., Gish W., Miller W., Myers E.W., Lipman D.J. (1990). Basic local alignment search tool. J. Mol. Biol..

[52] Reglinski M., Sriskandan S., Turner C.E. (2019). Identification of two new core chromosome-encoded superantigens in *Streptococcus pyogenes*; speQ and speR. J. Infect..

[53] Commons R.J., Smeesters P.R., Proft T., Fraser J.D., Robins-Browne R., Curtis N. (2014). Streptococcal superantigens: Categorization and clinical associations. Trends Mol. Med..

[54] Vega L.A., Malke H., McIver K.S. (2022). Virulence-Related Transcriptional Regulators of *Streptococcus pyogenes*. Streptococcus pyogenes: Basic Biology to Clinical Manifestations.

[55] Zorzoli A., Meyer B.H., Adair E., Torgov V.I., Veselovsky V.V., Danilov L.L., Uhrin D., Dorfmueller H.C. (2019). Group A, B, C, and G *Streptococcus* Lancefield antigen biosynthesis is initiated by a conserved alpha-d-GlcNAc-beta-1,4-l-rhamnosyltransferase. J. Biol. Chem..

[56] Florensa A.F., Kaas R.S., Clausen P., Aytan-Aktug D., Aarestrup F.M. (2022). ResFinder—An open online resource for identification of antimicrobial resistance genes in next-generation sequencing data and prediction of phenotypes from genotypes. Microb. Genom..

[57] Alcock B.P., Huynh W., Chalil R., Smith K.W., Raphenya A.R., Wlodarski M.A., Edalatmand A., Petkau A., Syed S.A., Tsang K.K. (2023). CARD 2023: Expanded curation, support for machine learning, and resistome prediction at the Comprehensive Antibiotic Resistance Database. Nucleic Acids Res..

[58] Bauer A.W., Perry D.M., Kirby W.M. (1959). Single-disk antibiotic-sensitivity testing of staphylococci; an analysis of technique and results. AMA Arch. Intern. Med..

[59] CLSI (2024). Performance Standards for Antimicrobial Disk and Dilution Susceptibility Tests for Bacteria Isolated From Animals.

[60] Rafailidis P.I., Kofteridis D. (2022). Proposed amendments regarding the definitions of multidrug-resistant and extensively drug-resistant bacteria. Expert. Rev. Anti Infect. Ther..

[61] Magiorakos A.P., Srinivasan A., Carey R.B., Carmeli Y., Falagas M.E., Giske C.G., Harbarth S., Hindler J.F., Kahlmeter G., Olsson-Liljequist B. (2012). Multidrug-resistant, extensively drug-resistant and pandrug-resistant bacteria: An international expert proposal for interim standard definitions for acquired resistance. Clin. Microbiol. Infect..

[62] Jelinkova J., Bícovă R., Rotta J. (1967). Some manifestations of a relationship between group A and L *streptococci*. Preliminary report. J. Hyg. Epidemiol. Microbiol. Immunol..

[63] Schaufuss P., Lämmler C., Niewerth B., Blobel H. (1987). Properties of L-*streptococci* in comparison with those of A-*streptococci*. Med. Microbiol. Immunol..

[64] CLSI (2025). Performance Standards for Antimicrobial Susceptibility Testing.

[65] Kaci A., Jonassen C.M., Skrede S., Sivertsen A., Steinbakk M., Oppegaard O., Norwegian Study Group on *Streptococcus dysgalactiae* (2023). Genomic epidemiology of *Streptococcus dysgalactiae* subsp. *equisimilis* strains causing invasive disease in Norway during 2018. Front. Microbiol..

[66] Hare T., Fry R., Orr A. (1942). First Impressions of the Bets Haemolytic *Streptocoecus* Infection of Swine. Vet. Rec..

[67] Jones J.E. (1976). The serological classification of *streptococci* isolated from diseased pigs. Br. Vet. J..

[68] Hommez J., Devriese L.A., Castryck F., Miry C. (1991). Beta-hemolytic *streptococci* from pigs: Bacteriological diagnosis. Zentralbl. Veterinarmed. B.

[69] Jacobson M., Berglund M., Pettersson M., Sandstrom M., Matti F., Sjolund M., Backhans A., Ytrehus B., Ekman S. (2025). Pathological and bacteriological findings in sows, finisher pigs, and piglets, being culled for lameness. Porcine Health Manag..

[70] Cinthi M., Massacci F.R., Coccitto S.N., Albini E., Cucco L., Orsini M., Simoni S., Giovanetti E., Brenciani A., Magistrali C.F. (2023). Characterization of a prophage and a defective integrative conjugative element carrying the *optrA* gene in linezolid-resistant *Streptococcus dysgalactiae* subsp. *equisimilis* isolates from pigs, Italy. J. Antimicrob. Chemother..

[71] Zoric M., Nilsson E., Lundeheim N., Wallgren P. (2009). Incidence of lameness and abrasions in piglets in identical farrowing pens with four different types of floor. Acta Vet. Scand..

[72] Katsumi M., Kataoka Y., Takahashi T., Kikuchi N., Hiramune T. (1998). Biochemical and serological examination of beta-hemolytic *streptococci* isolated from slaughtered pigs. J. Vet. Med. Sci..

[73] Chauhan R., Laddika L., Dinesh M., Maganbhai B.J., Malik S., Sahoo M., Qureshi S., Tiwari A.K. (2020). Isolation, Molecular Identification and Antibiogram of *Streptococcus dysgalactiae* Isolates Recovered from Pigs. Int. J. Curr. Microbiol. Appl. Sci..

[74] Glambek M., Skrede S., Sivertsen A., Kittang B.R., Kaci A., Jonassen C.M., Jorgensen H.J., Oppegaard O., Norwegian Study Group on *Streptococcus dysgalactiae* (2024). Antimicrobial resistance patterns in *Streptococcus dysgalactiae* in a One Health perspective. Front. Microbiol..

[75] Lu B., Fang Y., Huang L., Diao B., Du X., Kan B., Cui Y., Zhu F., Li D., Wang D. (2016). Molecular characterization and antibiotic resistance of clinical *Streptococcus dysgalactiae* subsp. *equisimilis* in Beijing, China. Infect. Genet. Evol..

[76] Shinohara K., Murase K., Tsuchido Y., Noguchi T., Yukawa S., Yamamoto M., Matsumura Y., Nakagawa I., Nagao M. (2023). Clonal Expansion of Multidrug-Resistant *Streptococcus dysgalactiae* Subspecies *equisimilis* Causing Bacteremia, Japan, 2005–2021. Emerg. Infect. Dis..

[77] Scicchitano D., Leuzzi D., Babbi G., Palladino G., Turroni S., Laczny C.C., Wilmes P., Correa F., Leekitcharoenphon P., Savojardo C. (2024). Dispersion of antimicrobial resistant bacteria in pig farms and in the surrounding environment. Anim. Microbiome.

[78] Monger X.C., Gilbert A.A., Saucier L., Vincent A.T. (2021). Antibiotic Resistance: From Pig to Meat. Antibiotics.

