# Supplementary material for: Streptococcus dysgalactiae subsp. equisimilis from Diseased Pigs Are Genetically Distinct from Human Strains and Associated with Multidrug Resistance
Source: Microorganisms. 2025 Dec 19;14(1):9. doi: 10.3390/microorganisms14010009 (PMC12843900; doi:10.3390/microorganisms14010009)
Supplement: Supplementary file 1 [file microorganisms-14-00009-s001.zip › FIGURE-S2.pdf]

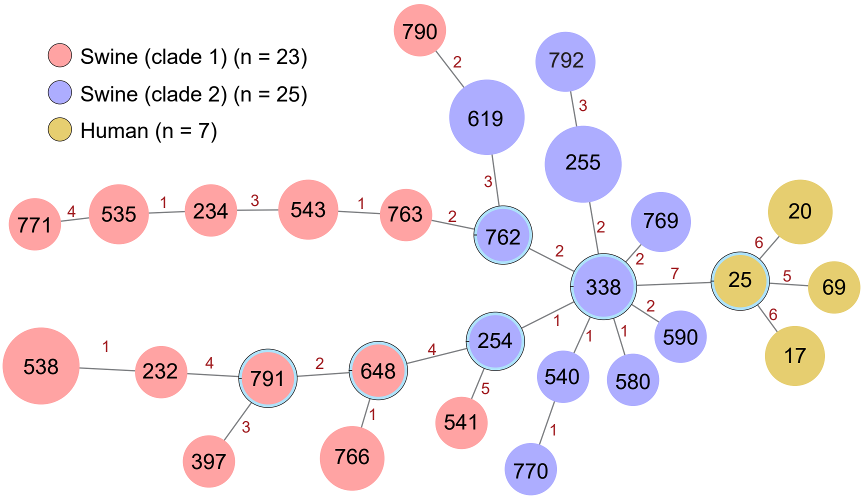

Figure S2. Minimum-spanning tree (MST) based on multilocus sequence typing (MLST) allelic profiles of *Streptococcus dysgalactiae* subsp. *equisimilis* (SDSE) isolates from swine and human infections. Each node represents a unique MLST allelic profile, with node size proportional to the number of isolates sharing that profile; numbers within nodes indicate sequence-type. Connecting lines denote inferred links between allelic profiles, with small numbers indicating the number of allele differences. The spatial layout is not drawn to scale. For swine isolates, clade designations correspond to those defined by the core-genome phylogenetic analysis in Figure 3.
