# Supplementary material for: Streptococcus dysgalactiae subsp. equisimilis from Diseased Pigs Are Genetically Distinct from Human Strains and Associated with Multidrug Resistance
Source: Microorganisms. 2025 Dec 19;14(1):9. doi: 10.3390/microorganisms14010009 (PMC12843900; doi:10.3390/microorganisms14010009)
Supplement: Supplementary file 1 [file microorganisms-14-00009-s001.zip › FIGURE-S3.pdf]

A

B

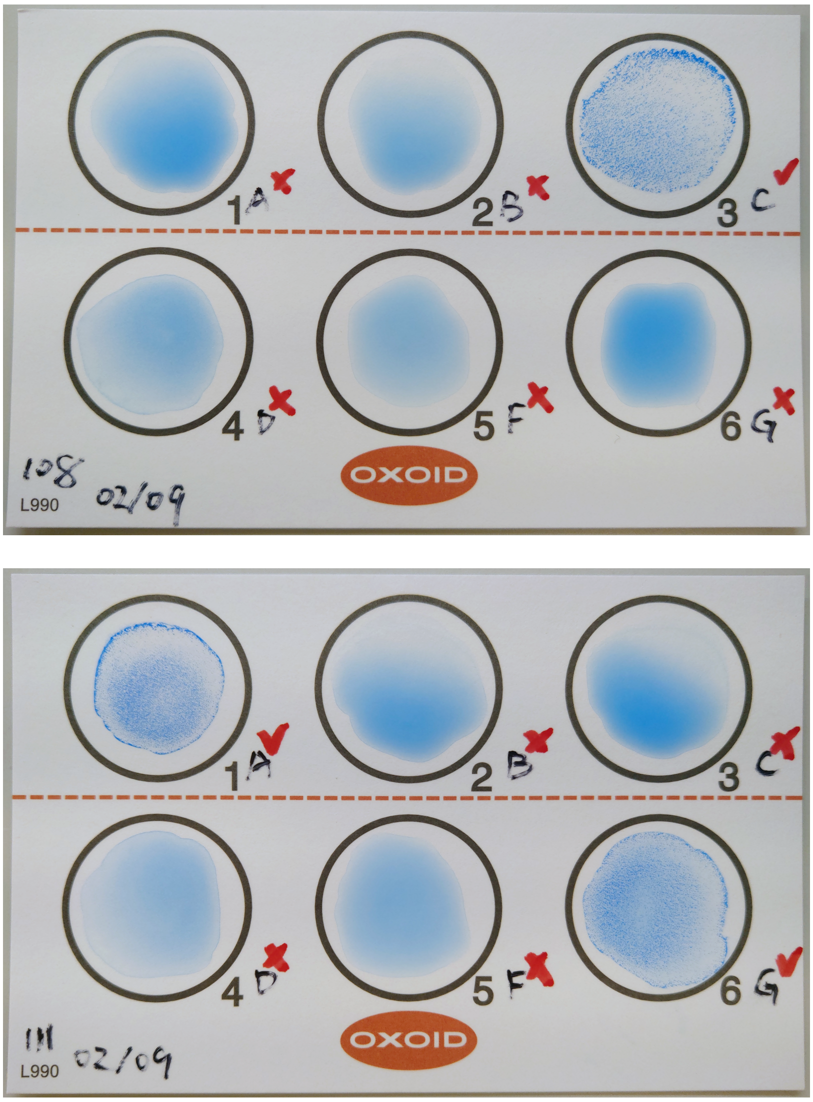

Figure S3. Lancefield grouping of two representative porcine *Streptococcus dysgalactiae* subsp. *equisimilis* (SDSE) isolates using the Oxoid latex agglutination kit. Circles 1 to 6 correspond to reactions with latex particles coated with Lancefield group A-, B-, C-, D-, F-, and G-specific antibodies, respectively. **(A)** NSDE00108 showed a single, strong agglutination reaction with group C antibody (circle 3). **(B)** NSDE00111 reacted with both group A and group G antibodies (circles 1 and 6), a cross-reaction pattern characteristic of group L isolates described in historical serological studies (see the main text).
