# Supplementary material for: Streptococcus dysgalactiae subsp. equisimilis from Diseased Pigs Are Genetically Distinct from Human Strains and Associated with Multidrug Resistance
Source: Microorganisms. 2025 Dec 19;14(1):9. doi: 10.3390/microorganisms14010009 (PMC12843900; doi:10.3390/microorganisms14010009)
Supplement: Supplementary file 1 [file microorganisms-14-00009-s001.zip › FIGURE-S1.pdf]

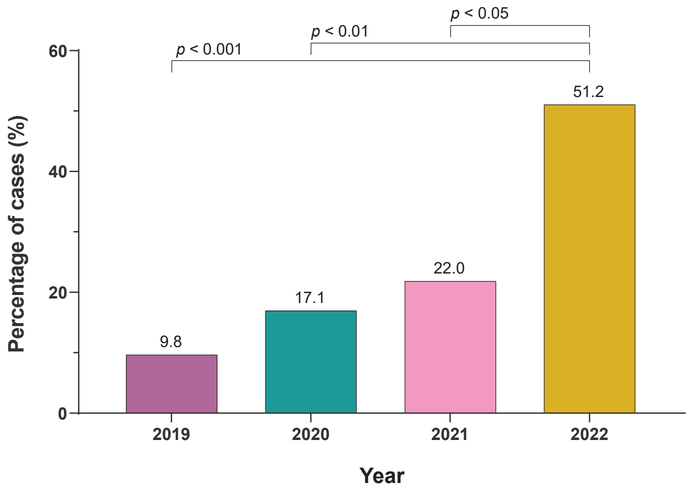

Figure S1. Annual distribution of *Streptococcus dysgalactiae* subsp. *equisimilis* (SDSE) cases identified in swine submissions from 2019 to 2022. Bars represent the percentage of SDSE-positive cases recorded each year. Statistical comparisons of proportions across years were performed using the χ^2 or Fisher's exact test, with significant differences indicated by *p*-values above the bars.
